# Efficacy and Safety of Daptomycin versus Vancomycin for Bacteremia Caused by Methicillin-Resistant *Staphylococcus* *aureus* with Vancomycin Minimum Inhibitory Concentration > 1 µg/mL: A Systematic Review and Meta-Analysis

**DOI:** 10.3390/pharmaceutics14040714

**Published:** 2022-03-27

**Authors:** Masaru Samura, Yuki Kitahiro, Sho Tashiro, Hiromu Moriyama, Yuna Hamamura, Isamu Takahata, Rina Kawabe, Yuki Enoki, Kazuaki Taguchi, Yoshio Takesue, Kazuaki Matsumoto

**Affiliations:** 1 Division of Pharmacodynamics, Keio University Faculty of Pharmacy, 1-5-30 Shibakoen, Minato-ku, Tokyo 105-8512, Japan; m.samura@hotmail.co.jp (M.S.); nikonikoyuki@keio.jp (Y.K.); s.t.try.medicinal@keio.jp (S.T.); hiro1015@keio.jp (H.M.); hamamura1997@keio.jp (Y.H.); sebass6060@gmail.com (I.T.); rina.0320-3-k.p@a6.keio.jp (R.K.); taguchi-kz@pha.keio.ac.jp (K.T.); matsumoto-kz@pha.keio.ac.jp (K.M.); 2 Department of Infection Control and Prevention, Hyogo College of Medicine, 1-1 Mukogawa-cho, Nishinomiya, Hyogo 663-8501, Japan; takesuey@hyo-med.ac.jp; 3 Department of Infectious Diseases, Tokoname City Hospital, 3-3-3 Asukadai, Tokoname, Aichi 479-8510, Japan

**Keywords:** daptomycin, vancomycin, methicillin-resistant *Staphylococcus aureus*, meta-analysis

## Abstract

This systematic review and meta-analysis compares the efficacy of daptomycin and vancomycin in adult patients with bacteremia by methicillin-resistant *Staphylococcus aureus* (MRSA) with vancomycin minimum inhibitory concentration (MIC) > 1 µg/mL. We searched the PubMed, Web of Science, Cochrane Library, and ClinicalTrials.gov databases on 12 May 2020. All-cause mortality (primary outcome) and treatment success rates were compared and subgroups stratified by infection source risk level and method of vancomycin susceptibility testing were also analyzed. Seven studies (*n* = 907 patients) were included in this efficacy analysis. Compared with vancomycin, daptomycin treatment was associated with significantly lower mortality (six studies, odds ratio (OR) 0.53, 95% confidence interval (CI) 0.29–0.98) and higher treatment success (six studies, OR 2.20, 95% CI 1.63–2.96), which was consistent regardless of the vancomycin MIC test method used. For intermediate-risk sources, daptomycin was a factor increasing treatment success compared with vancomycin (OR 4.40, 95% CI 2.06–9.40), and it exhibited a trend toward a higher treatment success rate for high-risk sources. In conclusion, daptomycin should be considered for the treatment of bacteremia caused by MRSA with vancomycin MIC > 1 µg/mL, especially in patients with intermediate- and high-risk bacteremia sources.

## 1. Introduction

Methicillin-resistant *Staphylococcus aureus* (MRSA) is a major cause of serious infections, including nosocomial bacteremia [1]. The mortality rate of patients with MRSA bacteremia is high, with estimates ranging from 30% to 50% [2]. This rate has been reported to exceed that of patients with bacteremia caused by methicillin-susceptible *S. aureus* [3,4]. The glycopeptide drug vancomycin (VCM) has been a mainstay of MRSA treatment for many years [5,6,7]. The Clinical and Laboratory Standards Institute established the VCM minimum inhibitory concentration (MIC) susceptibility breakpoint as 2 μg/mL for *S. aureus* while the definitions of vancomycin-intermediate *S. aureus* and vancomycin-resistant *S. aureus* were MICs of 4 to 8 μg/mL and ≥16 μg/mL, respectively [8]. Several reports have demonstrated that VCM is less effective against serious MRSA infections, with MIC values at the higher end of the susceptibility range [9,10,11]. A recent systematic review and meta-analysis by Ishaq et al. compared patients with MRSA caused by isolates with a high but susceptible VCM MIC (≥1.5 μg/mL) and a low MIC (<1.5 μg/mL); this study concluded that high MICs were associated with significantly increased mortality [11]. Although a phenomenon of gradual increase in the value of VCM MICs for MRSA was previously reported [12], a systematic review conducted by Diaz et al. did not detect VCM MIC creep [13]. Hsu et al. reported a frequency of 7% for MRSA isolates with VCM MIC > 1 µg/mL, as determined by the reference broth microdilution (BMD) method [14].

Guidelines for the treatment of MRSA infections by the Infectious Diseases Society of America recommend that the continued use of VCM for MRSA isolates with VCM MICs ≤ 2 μg/mL should be guided by the patient’s clinical response, independently of the MIC [5]. However, alternate regimens should be clearly shown to result in better clinical outcomes in sepsis patients with MRSA infections caused by isolates with VCM MICs > 1 µg/mL.

Daptomycin (DAP) is a lipopeptide drug used to treat MRSA infections. Several studies have compared the efficacy and safety of VCM and DAP in patients with MRSA bacteremia [15,16,17,18,19,20,21]. A recent meta-analysis by Maraolo et al. demonstrated that DAP was associated with a significantly reduced risk of clinical failure compared with VCM in patients with MRSA bacteremia [22]. However, of the eight studies evaluated in that meta-analysis, five evaluated only patients with MRSA bacteremia with VCM MICs > 1 µg/mL, which might have influenced the results. The aim of the present study is to perform a systematic review and meta-analysis comparing the clinical outcomes of patients treated with VCM versus DAP for MRSA bacteremia with VCM MICs > 1 µg/mL, with special emphasis on all-cause mortality as the primary efficacy outcome. Additional subgroup analyses are performed to compare mortality and treatment success according to the bacteremia source risk category and VCM MIC test methodology.

## 2. Materials and Methods

### 2.1. Search Strategy and Study Selection Criteria

We conducted our study following the Preferred Reporting Items for Systematic reviews and Meta-analyses guidelines [23]. We performed a literature search using four electronic databases: PubMed, Web of Science, the Cochrane Library, and ClinicalTrials.gov on 12 May 2020. Three authors independently searched the literature using the search terms listed in Tables S1–S4 and screened the articles to exclude duplicates. The inclusion criteria for the studies were: (1) comparison of the efficacy and safety of DAP and VCM; (2) MRSA bacteremia with VCM MICs > 1 µg/mL; and (3) patient age ≥ 18 years old. The exclusion criteria were: (1) VCM administered in combination with gentamicin; (2) anti-MRSA drugs other than DAP and VCM administered in combination; (3) infections caused by pathogens other than MRSA; and (4) insufficient data for efficacy comparison. Five authors independently conducted the screening procedures, and, in cases of differing opinions, the findings were discussed with another author until a consensus was reached.

### 2.2. Data Extraction and Subgroup Categories

Three authors independently extracted the data from each study. In case of a differing opinion, a consensus was reached through discussion. The study design, study duration, country, age, total number of patients, MIC testing method, dose regimen, and source of bacteremia were extracted from each study. We analyzed all-cause mortality as the primary efficacy outcome. In addition, treatment success rate, recurrence rate, and rehospitalization rate were analyzed as the secondary efficacy outcomes. Treatment success was evaluated based on clinical success and composite failure rates. In studies that reported a composite failure rate, the number of composite failures was subtracted from the overall number of patients and was used as the clinical success rate.

We performed subgroup analyses to compare mortality and treatment success efficacy outcomes according to the bacteremia source risk level. For this, the infection source was assigned to one of three categories, as described for previous studies [24,25]: low-risk sources (mortality < 10%) included intravenous catheter, urinary tract, ear–nose–larynx, gynecological, and various manipulation-related sources; intermediate-risk sources (mortality 10–20%) included osteoarticular, soft tissue, and unknown sources; and high-risk sources (mortality > 20%) included intravascular (e.g., endocarditis), abdominal, and central nervous system sources. Another subgroup analysis was performed to compare the VCM MIC testing methodology: the E-test or Microscan versus the BMD method, as described in a previous study [26]. Recurrence and rehospitalization rates were analyzed for studies with available data. Safety was evaluated by extracting data on adverse drug reactions in each article.

The incidences of nephrotoxicity and increased serum creatine phosphokinase (CPK) levels were evaluated as the primary safety outcome in patients treated with VCM and DAP. Nephrotoxicity was defined as an increase in serum creatinine of ≥0.5 µmol/L or ≥50% from baseline. The incidence of CPK elevation was evaluated for studies that reported serum CPK levels exceeding five times the upper limit of normal or >500 U/L.

### 2.3. Statistical Analysis

We performed the meta-analysis using Review Manager for Windows (RevMan, Version 5.4, Copenhagen, Denmark; The Nordic Cochrane Center, The Cochrane Collaboration, 2020) and constructed forest plots. Odds ratios (ORs) and 95% confidence intervals (CIs) were calculated using the Mantel–Haenszel method and a random effect model. Statistical heterogeneity among studies was assessed using the *I*^2^ statistic. *I*^2^ values of ≥50%, 25–50%, and ≤25% were regarded as strong, moderate, and no heterogeneity, respectively. As a sensitivity analysis, the quality of each article was scrutinized, and, if a high degree of heterogeneity was detected, an analysis excluding reports that contributed to the heterogeneity was conducted. We assessed publication bias via funnel plots, Begg’s rank correlation test, and Egger’s weighted regression methods. We used R version 4.0.2 to conduct Begg’s rank correlation test and Egger’s weighted regression methods. *P*-values have been reported, with hypothesis testing set at the two-tailed significance level of <0.05.

## 3. Results

### 3.1. Search Results

**Figure 1:**
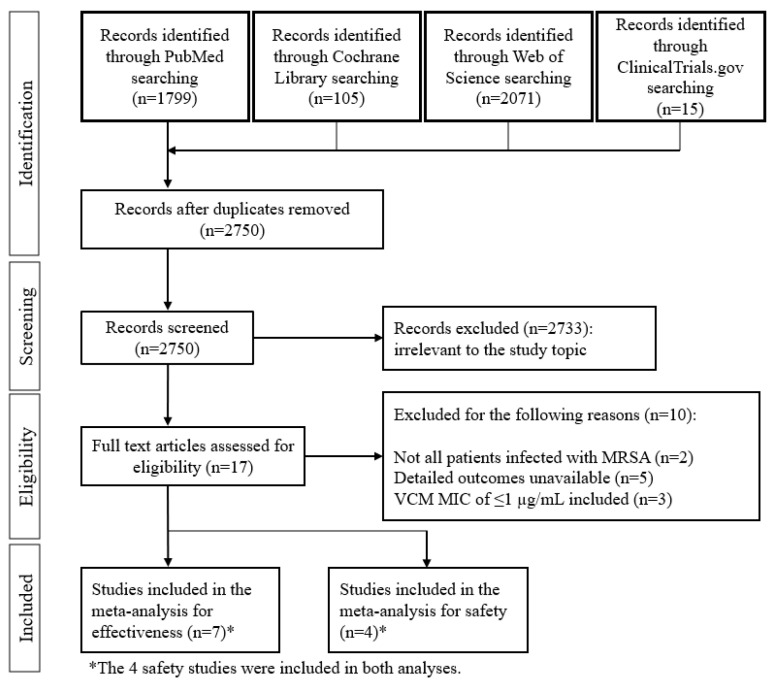
Flow chart of the study selection process.

The database search of studies evaluating the efficacy and safety of DAP and VCM against MRSA bacteremia identified a total of 3990 articles for screening (Figure 1).

Of these 3990, 17 articles satisfied the inclusion criteria, and an additional 10 studies were removed based on the exclusion criteria. In total, the final meta-analysis for efficacy evaluation included seven studies [15,16,17,20,21,27,28], of which four were also included in the meta-analysis of safety [20,21,27,28]. The characteristics of the seven included studies are shown in Table 1. The studies by Cubist (2018) and Kalimuddin (2018) were prospective randomized control trials (RCTs), and the remaining five were retrospective studies. Testing of VCM MIC was performed by BMD and the other methods in one and five studies, respectively. Only the Cubist (2018) study did not indicate the testing method of MIC. Data from studies that employed more than one test were included in each subgroup. The doses of DAP administered were 6 mg/kg every 24 h (q24h) in the two RCTs and ≥6 mg/kg q24h in the five retrospective studies. A loading dose of VCM was included in one study (Cheng 2013), and the maintenance dose was 15–20 mg/kg in three studies. The target VCM trough concentrations were between 10 and 20 µg/mL in all seven studies. None of the studies employed area under the time-concentration curve (AUC)-guided dosing.

### 3.2. Analysis of the Mortality and Treatment Success of DAP versus VCM for Bacteremia Caused by MRSA with VCM MIC > 1 µg/mL

**Figure 2:**
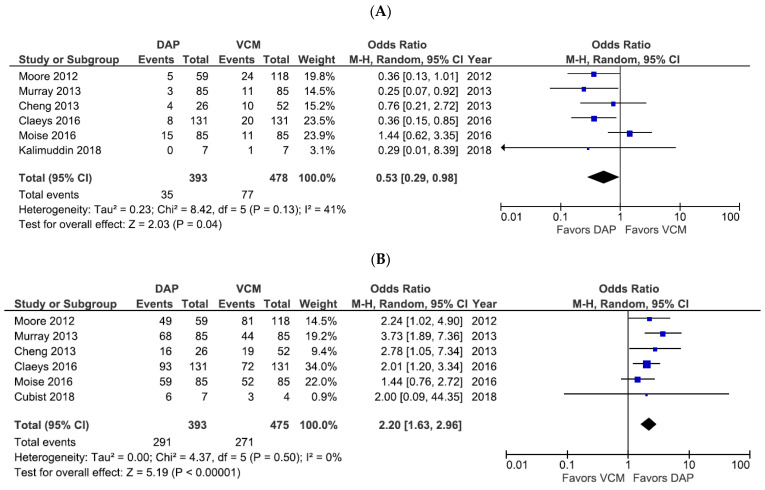
Forest plots of all-cause mortality and treatment success rates for patients treated with DAP versus VCM for bacteremia caused by MRSA with VCM MIC > 1 µg/mL. (**A**,**B**) Forest plots of the all-cause mortality rate (**A**) and treatment success rate (**B**) of patients treated with DAP versus VCM in the studies evaluated. The vertical line indicates no significant difference between the groups compared. Diamonds and horizontal lines represent the Mantel–Haenszel ORs and 95% CIs, respectively. Squares indicate point estimates, and the size of the square indicates the weight of each study included in the meta-analysis. CI, confidence interval; DAP, daptomycin; MIC, minimum inhibitory concentration; MRSA, methicillin-resistant *Staphylococcus aureus*; OR, odds ratio; VCM, vancomycin.

Six studies included all-cause mortality as the primary efficacy outcome (Figure 2A), of which three studies evaluated 30-day mortality and three evaluated 60-day mortality. Among the six studies, DAP treatment was associated with a significantly lower risk of all-cause mortality compared with VCM (OR 0.53, 95% CI 0.29–0.98, *p* = 0.04, *I*^2^ = 41%; Figure 2A). The results of the independent 30-day mortality (OR 0.40, 95% CI 0.21–0.74, *p* = 0.004, *I*^2^ = 0%) and 60-day mortality (OR 0.68, 95% CI 0.22–2.14, *p* = 0.52, *I*^2^ = 56%) analyses are shown in Figure S1. The six studies that compared treatment success rates as a secondary efficacy outcome showed that DAP had a significantly higher treatment success rate than VCM (OR 2.20, 95% CI 1.63–2.96, *p* < 0.00001, *I*^2^ = 0%; Figure 2B).

**Figure 3:**
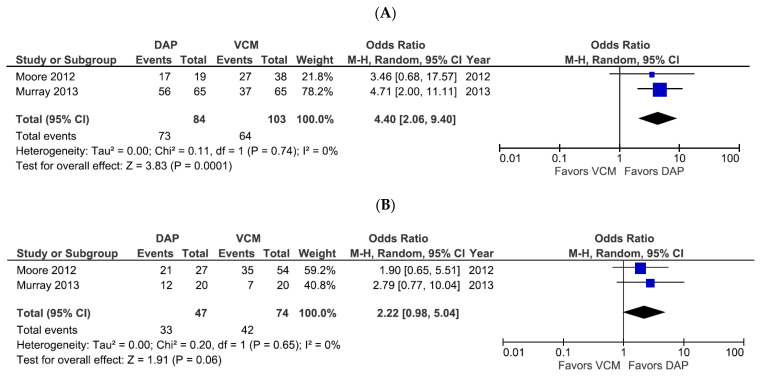
Forest plots of treatment success rates for patients treated with DAP versus VCM for bacteremia caused by MRSA with VCM MIC > 1 µg/mL, according to the infection risk level. (**A**,**B**) Forest plots of treatment success rates of patients treated with DAP versus VCM for intermediate-risk (**A**) and high-risk (**B**) sources of infection. Symbols and abbreviations are as described in Figure 2.

We further examined treatment success rates for DAP- and VCM-treated patients according to the MRSA bacteremia source risk level (Figure 3). Only one study evaluated the treatment success rate in low-risk source infections, and it showed that DAP did not increase the success rate compared to VCM (OR 2.03, 95% CI 0.36–11.52, *p* = 0.43). However, the two studies that evaluated patients with intermediate-risk and high-risk bacteremia sources showed that DAP had a significantly higher treatment success rate than VCM for intermediate-risk sources (OR 4.40, 95% CI 2.06–9.40, *p* = 0.0001, *I*^2^ = 0%; Figure 3A) and exhibited a trend toward a higher treatment success rate for high-risk sources, although the difference narrowly missed reaching statistical significance (OR 2.22, 95% CI 0.98–5.04, *p* = 0.06, *I*^2^ = 0%; Figure 3B). None of the studies included data on mortality rates based on bacteremia source risk level.

In subgroup analyses based on the VCM MIC testing method, we detected no significant difference in the mortality rates of DAP- and VCM-treated patients when the VCM was measured using E-test or Microscan (OR 0.60, 95% CI 0.26–1.37, *p* = 0.22, *I*^2^ = 56%; Figure S2). Post-hoc sensitivity analysis demonstrated that the incoherence was eliminated after excluding the study by Moise et al., which found that the proportion of infection sources with a high risk of death, such as bone or joint involvement and endocarditis, were higher in the DAP group.

**Figure 4:**
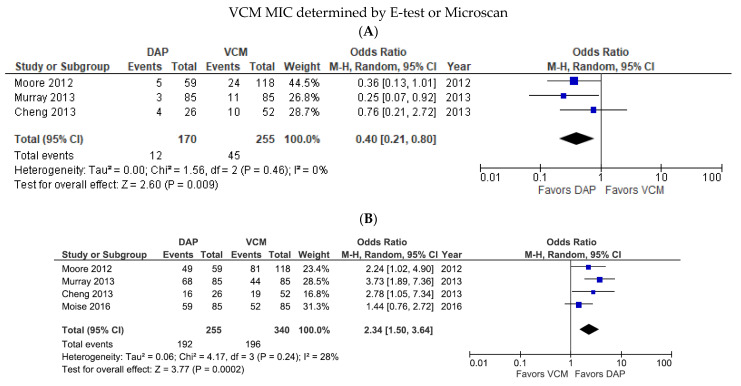
Forest plots of the mortality and treatment success rates of patients treated with DAP versus VCM for bacteremia caused by MRSA with VCM MIC > 1 µg/mL, according to the MIC test method. (**A**,**B**) Forest plots of the mortality rate (**A**) and treatment success rate (**B**) of DAP versus VCM when the VCM MIC was determined by E-test or Microscan. Symbols and abbreviations are as described in Figure 2.

The mortality rates were higher for DAP-treated patients than for VCM-treated patients in this subgroup (OR = 0.40, 95% CI = 0.21–0.80, *p* = 0.009, *I*^2^ = 0%; Figure 4A), whereas the treatment success rate was higher for DAP-treated than VCM-treated patients in this subgroup (OR 2.34, 95% CI 1.50–3.64, *p* = 0.0002, *I*^2^ = 28%; Figure 4B). One study tested VCM MIC using the BMD method, and that study showed significantly better outcomes for DAP than VCM with respect to both mortality rate (OR 0.36, 95% CI 0.15–0.85, *p* = 0.02; data not shown) and treatment success (OR 2.01, 95% CI 1.20–3.34, *p* = 0.008; data not shown).

Data allowing comparisons of recurrence rates were available for five articles and showed no significant difference between patients treated with DAP and VCM (OR 0.38, 95% CI 0.11–1.26, *p* = 0.11, *I^2^* = 0%; Figure S3A). Similarly, there was no significant difference in the rehospitalization rates of DAP- and VCM-treated patients based on the two studies with relevant data (OR 0.97, 95% CI 0.58–1.61, *p* = 0.91, *I^2^* = 0%; Figure S3B).

### 3.3. Safety Analysis of DAP versus VCM Treatment for Bacteremia Caused by MRSA with VCM MIC > 1 µg/mL

**Figure 5:**
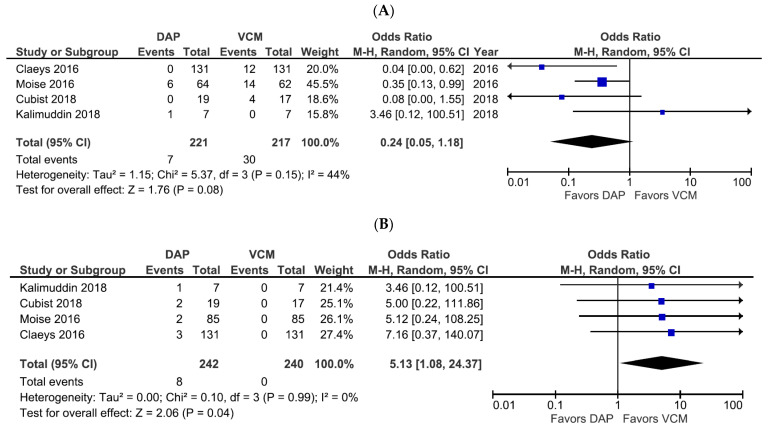
Forest plots of the safety of DAP versus VCM for the treatment of patients with bacteremia caused by MRSA with VCM MIC > 1 µg/mL. (**A**,**B**) Forest plots of the incidence of nephrotoxicity (**A**) and CPK elevation (**B**) in patients treated with DAP versus VCM in the studies evaluated. Symbols and abbreviations are as described in Figure 2.

Adverse drug reactions (comprising CPK elevation, nephrotoxicity, skin symptoms, and gastrointestinal symptoms) were extracted from six studies with available data (Table S5). In the four studies containing nephrotoxicity and CPK elevation data as the primary safety outcome, a trend towards a lower incidence of nephrotoxicity with DAP was observed, albeit not significant (OR 0.24, 95% CI 0.05–1.18, *p* = 0.08, *I*^2^ = 44%; Figure 5A). However, DAP treatment was associated with a significantly higher incidence of CPK elevation compared with VCM treatment (OR 5.13, 95% CI 1.08–24.37, *p* = 0.04, *I*^2^ = 0%; Figure 5B).

### 3.4. Assessment of Publication Bias

**Figure 6:**
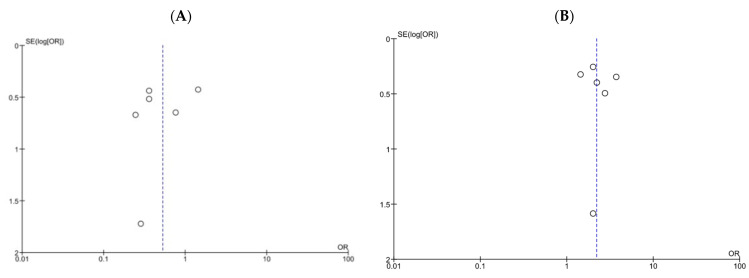
Funnel plots of all-cause mortality and treatment success for patients treated with DAP versus VCM for bacteremia caused by MRSA with VCM MIC > 1 µg/mL. (**A**,**B**) Funnel plots of all-cause mortality (**A**) and treatment success (**B**). The dashed lines represent pooled ORs of 0.53 (**A**) and 2.20 (**B**), respectively. SE, standard error; OR, odds ratio.

We assessed publication bias using funnel plots of all-cause mortality (Figure 6A) and treatment success (Figure 6B), Begg’s rank correlation test (corrected *p* = 0.72 and 0.72, respectively), and Egger’s estimated bias coefficient (−1.10; *p* = 0.55 and 0.38; *p* = 0.76, respectively) in all-cause mortality and treatment success. No marked publication bias was observed in both analyses.

## 4. Discussion

The meta-analysis, including 13 RCTs of any type of infection, did not demonstrate significant differences in efficacy and mortality between DAP and a comparator regimen, which was VCM in 10 of the 13 RCTs [29]. However, the present meta-analysis of patients with bacteremia caused by MRSA with VCM MIC > 1 µg/mL demonstrated a significantly lower mortality rate and higher treatment success rate for DAP than for VCM. Although several differences in inclusion criteria among the included studies should be considered, the present meta-analysis demonstrates the superiority of DAP over VCM, especially for patients with bacteremia caused by MRSA with VCM MIC > 1 µg/mL, in which poor outcomes were reported for VCM therapy.

The treatment strategy might differ between uncomplicated and complicated bacteremia, such as bacteremia caused by infective endocarditis, cardiac device infection, osteomyelitis, septic arthritis, and pneumonia [30]. The selection of an appropriate antibiotic as the first-line therapy, rather than changing antibiotics based on the clinical course, is essential to improve the outcomes of patients with serious infections, including sepsis/septic shock [31]. Hence, we compared clinical outcomes between DAP and VCM according to the MRSA bacteremia source risk level, and favorable trends for DAP were observed in patients with intermediate-risk sources, such as osteoarticular infection, and high-risk sources, such as endocarditis in bacteremia caused by VCM MIC > 1 µg/mL strains. However, the higher treatment success rate for DAP compared to VCM was not confirmed in patients with low-risk sources of bacteremia, such as removable intravenous catheters. Recent guidelines indicate that DAP should be used at doses of >6 mg/kg in patients with infective endocarditis or bone infection caused by MRSA [5,6,7]. Several studies have suggested that DAP should be administered at doses of >6 mg/kg to achieve AUC/MIC ≥ 666 as a measure of treatment efficacy [32,33,34,35,36]. Doses exceeding 6 mg/kg were used in all but one study included in our meta-analysis.

In the present study, the incidence of nephrotoxicity tended to be higher in patients treated with VCM than in those treated with DAP. We excluded a study in which the VCM arm permitted gentamicin combination therapy [37], which might increase the risk of nephrotoxicity [38,39]. The risk factors reported for VCM-induced nephrotoxicity include trough-guided dosing, in which the target trough concentrations are 15–20 µg/mL [40]. The upper threshold of the target trough range was 20 µg/mL in six of the seven selected studies in the present meta-analysis, which may have had a considerable impact on the results. The rate of CPK elevation was significantly higher in patients treated with DAP than with VCM. Trough concentrations of ≥19.5 or 24.3 µg/mL have been demonstrated as risk factors for CPK elevation upon treatment with DAP [41,42,43,44].

There are several limitations to the present study. First, although adjustment for potential confounding factors using propensity score matching or matched case-control was conducted, five of the seven studies selected for the meta-analysis were retrospective. A considerable number of patients in the DAP group were switched from VCM, which may have influenced the results. Second, we did not prospectively register the study in a systematic review protocol in an international database. However, as we have conducted our study in accordance with PRISMA2020, we consider the results of the study to be valid. Third, because no negative findings were reported, publication bias should be considered. Fourth, our meta-analysis did not include studies that evaluated the efficacy and safety of VCM based on AUC-guided dosing according to the latest recommendations [40,45,46]. Trough-guided dosing tended to increase the risk of nephrotoxicity more strongly than AUC-guided dosing in the meta-analysis [40]. Fifth, only one study used a regimen that included a loading dose. Casapao et al. demonstrated that early VCM exposure increased the treatment success in patients with MRSA infective endocarditis [47]. A VCM loading dose is essential to achieve an early target concentration [6,45]. Sixth, patients with MRSA bacteremia tend to have complications such as infective endocarditis, osteomyelitis, abscesses, and prosthetic-device-related infections, which often require non-pharmacological treatments such as surgery, drainage, and device removal [5,6,7]. These factors may have had a significant impact on the results. Seventh, DAP has been shown to be inactivated by pulmonary surfactants [48] and is, therefore, not recommended for the treatment of pneumonia [6]. Six of seven studies included in our meta-analysis excluded patients with pneumonia as the source of bacteremia, but it is unclear whether that was also the case for the study by Cheng et al. [16]. Finally, MIC values obtained by E-test are generally higher than those obtained by the BMD method, and only one study in our analysis employed BMD for MIC testing [20]. However, the BMD study included a substantial number of patients (262), and DAP was a significantly more effective treatment than VCM in that study [20], which might support the conclusion even though it was one study.

## 5. Conclusions

Our systematic review and meta-analysis suggest DAP may be preferable as the first choice of antibiotic for the treatment of intermediate- and high-risk sources of bacteremia by MRSA with VCM MIC > 1 µg/mL. Although there is the caveat that the majority of included studies determined MIC by the E-test and Microscan method, the higher treatment success rate with daptomycin compared to VCM was consistent, regardless of the VCM MIC test method used.

## Figures and Tables

**Table 1:** Characteristics of studies included in the meta-analysis.

Study	Study Design	Duration	Age (years)	Country	No. of Patients		Vancomycin MIC > 1 μg/mL			Dosage Regimen		Bacteremia Source	
					DAP	VCM	Test Criteria	No. of Patients		DAP	VCM	DAP	VCM
								DAP	VCM				
Moore 2012 [15]	Retrospective case-control	2005–2009	≥18	USA	59	118	E-test 1.5 μg/mL 2 μg/mL	25 34	102 16	6 mg/kg q24h	Target C_min_ 10*–*20 µg/mL	CRBSI 17%; IE 29%; SSTI 32%; IAI 3%; Genitourinary 5%; Graft/device: 10%; Other 3%	CRBSI:22%; IE: 29%; SSTI 28%; IAI 4%; Genitourinary 0%; Graft/device: 9%; Other 7%
Cheng 2013 [16]	Retrospective case-control	2009–2010	≥18	Taiwan	26	52	E-test ≥1.5 μg/mL	26	52	8*–*10 mg/kg q24h	Loading 25*–*30 mg/kg; maintenance 15*–*20 mg/kg q12h	No data	No data
Murray 2013 [17]	Retrospective cohort	2005–2012	Adult	USA	85	85	1st stage: E-test; 2nd stage Microscan >1 μg/mL	85	85	≥6 mg/kg q24h	Target C_min_ 15*–*20 µg/mL	IE 23.5%; B/J 34.1%; SSTI 32.9%; unknown 9.4%	IE: 23.5%; B/J 34.1%; SSTI 32.9%; unknown 9.4%
Claeys 2016 [20]	Retrospective cohort	2010–2015	≥18	USA	131	131	BMD >1 μg/mL	131	131	Median 8.2 mg/kg q24h (IQR, 6.4*–*10.0)	C_min_: median 17.7 mg/L (IQR, 13.2*–*22.0)	B/J 29.0%; SSTI 22.9%; deep abscess: 10.7%; IE: 19.1%; CRBSI 7.6%; others 10.7%	B/J 20.6%; SSTI 25.2%; deep abscess: 10.7%; IE 17.6%; CRBSI 11.5%; others 14.5%
Moise 2016 [21]	Retrospective cohort	No data	≥18	USA	85	85	E-test: 61%; Microscan: 27% Phoenix: 12% 1.5 μg/mL 2 μg/mL	27 58	41 44	Median 6 mg/kg q24h (IQR, 6–8)	C_min_: median 17.5 mg/L (IQR, 14.0–22.0)	IE 24%; infected aneurysm 2%; septic thrombophlebitis 1%; unknown 12%; B/J 32%; SSTI 21%; IAI/UTI 2%; CRBSI 6%	IE 13%; infected aneurysm 4%; septic thrombophlebitis 11%; unknown 12%; B/J 18%; SSTI 29%; IAI/UTI 8%; CRBSI: 6%
Cubist 2018 [27]	RCT	2008–2010	≥18	USA	19	17	No data >1 μg/mL	19 (100)	17 (100)	10 mg/kg q24h	15 mg/kg q12h; target trough 15–20 µg/mL	Bacteremia W/O IE	Bacteremia W/O IE
Kalimuddin 2018 [28]	RCT	2014–2015	≥21	Singapore	7	7	E-test or VITEK2 1.5 ≤ MIC < 2	7 (100)	7 (100)	6 or 8 mg/kg q24h	15 mg/kg q12h; target C_min_ 15–20 µg/mL	Bacteremia uncomplicated: 71.4% complicated (without IE) 28.6%	Bacteremia uncomplicated: 100% complicated (without IE) 0%

MIC, minimum inhibitory concentration; BMD, broth microdilution; VTEK2, automated instrument for microbial identification and antibiotic susceptibility testing; RCT, randomized controlled trial; C_min_, trough concentration; DAP, daptomycin; VCM, vancomycin; IQR, interquartile range; IE, infective endocarditis; CRBSI, catheter-related bloodstream infection; B/J, bone /joint infection; SSTI, skin and soft tissue infection; IAI, intraabdominal infection; UTI, urinary tract infection.

## Data Availability

**T**he data presented in this study are available upon request from the corresponding author.

## Supplementary Materials

The following supporting information can be downloaded at: https://www.mdpi.com/article/10.3390/pharmaceutics14040714/s1, Figure S1: Forest plots of the 30-day and 60-day mortality rates for patients treated with DAP versus VCM for bacteremia caused by MRSA with VCM MIC > 1 µg/ML, Figure S2: Forest plots of the mortality rates of patients treated with DAP versus VCM for bacteremia caused by MRSA with VCM MIC > 1 µg/mL, determined by E-test or Microscan, Figure S3: Forest plots of the recurrence and rehospitalization rates for patients treated with DAP versus VCM for bacteremia caused by MRSA with VCM MIC > 1 µg/mL, Table S1: Terms used in and results of searches of PubMed, Table S2: Terms used in and results of searches of Web of Science, Table S3: Terms used in and results of searches of the Cochrane Library, Table S4: Terms used in and results of searches of ClinicalTrials.gov, Table S5 Adverse drug reactions with DAP and VCM in selected studies.
