# Supplementary material for: Efficacy and Safety of Daptomycin versus Vancomycin for Bacteremia Caused by Methicillin-Resistant Staphylococcus aureus with Vancomycin Minimum Inhibitory Concentration > 1 µg/mL: A Systematic Review and Meta-Analysis
Source: Pharmaceutics. 2022 Mar 27;14(4):714. doi: 10.3390/pharmaceutics14040714 (PMC9032134; doi:10.3390/pharmaceutics14040714)
Supplement: Supplementary file 1 [file pharmaceutics-14-00714-s001.zip › pharmaceutics-1540606-supplementary.pdf]

pharmaceutics

MDPI

Supplementary Materials: Efficacy and Safety of Daptomycin versus Vancomycin for Bacteremia Caused by Methicillin-Resistant *Staphylococcus aureus* with Vancomycin Minimum Inhibitory Concentration >1 μg/mL: A Systematic Review and Meta-Analysis

Masaru Samura, Yuki Kitahiro, Sho Tashiro, Hiromu Moriyama, Yuna Hamamura, Isamu Takahata, Rina Kawabe, Yuki Enoki *, Kazuaki Taguchi, Yoshio Takesue and Kazuaki Matsumoto

(A)

	DAP		VCM			Odds Ratio	
Study or Subgroup	Events	Total	Events	Total	Weight	M-H, Random, 95% CI	Year
Murray 2013	3	85	11	85	22.7%	0.25 [0.07, 0.92]	2013
Cheng 2013	4	26	10	52	24.3%	0.76 [0.21, 2.72]	2013
Claeys 2016	8	131	20	131	53.0%	0.36 [0.15, 0.85]	2016
**Total (95% CI)**		**242**		**268**	**100.0%**	**0.40 [0.21, 0.74]**	
Total events	15		41				

Heterogeneity: Tau² = 0.00; Chi² = 1.58, df = 2 (P = 0.45); I² = 0%
Test for overall effect: Z = 2.89 (P = 0.004)

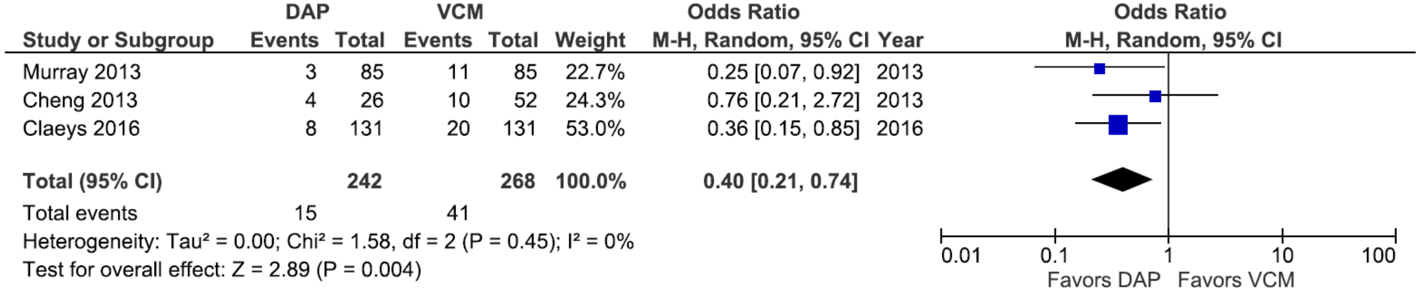

(B)

Study or Subgroup	DAP Events	DAP Total	VCM Events	VCM Total	Weight	Odds Ratio M-H, Random, 95% CI	Year
Moore 2012	5	59	24	118	42.6%	0.36 [0.13, 1.01]	2012
Moise 2016	15	85	11	85	47.7%	1.44 [0.62, 3.35]	2016
Kalimuddin 2018	0	7	1	7	9.7%	0.29 [0.01, 8.39]	2018
**Total (95% CI)**		**151**		**210**	**100.0%**	**0.68 [0.22, 2.14]**	
Total events	20		36				

Heterogeneity: Tau² = 0.53; Chi² = 4.56, df = 2 (P = 0.10); I² = 56%
Test for overall effect: Z = 0.65 (P = 0.52)

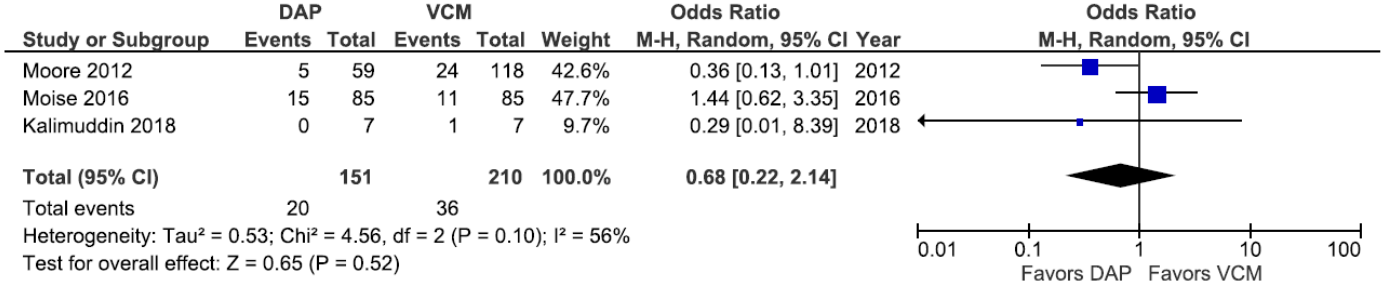

Figure S1. Forest plots of the 30-day and 60-day mortality rates for patients treated with DAP *versus* VCM for bacteremia caused by MRSA with VCM MIC > 1 μg/mL.

(A and B) Forest plots of the 30-day mortality rate (A) and 60-day mortality rate (B) of patients treated with DAP *versus* VCM in the studies evaluated. Symbols and abbreviations are as described for Figure 2.

Study or Subgroup	DAP Events	DAP Total	VCM Events	VCM Total	Weight	Odds Ratio M-H, Random, 95% CI	Year
Moore 2012	5	59	24	118	26.6%	0.36 [0.13, 1.01]	2012
Murray 2013	3	85	11	85	21.0%	0.25 [0.07, 0.92]	2013
Cheng 2013	4	26	10	52	21.8%	0.76 [0.21, 2.72]	2013
Moise 2016	15	85	11	85	30.6%	1.44 [0.62, 3.35]	2016
**Total (95% CI)**		**255**		**340**	**100.0%**	**0.60 [0.26, 1.37]**	
Total events	27		56				

Heterogeneity: Tau² = 0.39; Chi² = 6.87, df = 3 (P = 0.08); I² = 56%
Test for overall effect: Z = 1.21 (P = 0.22)

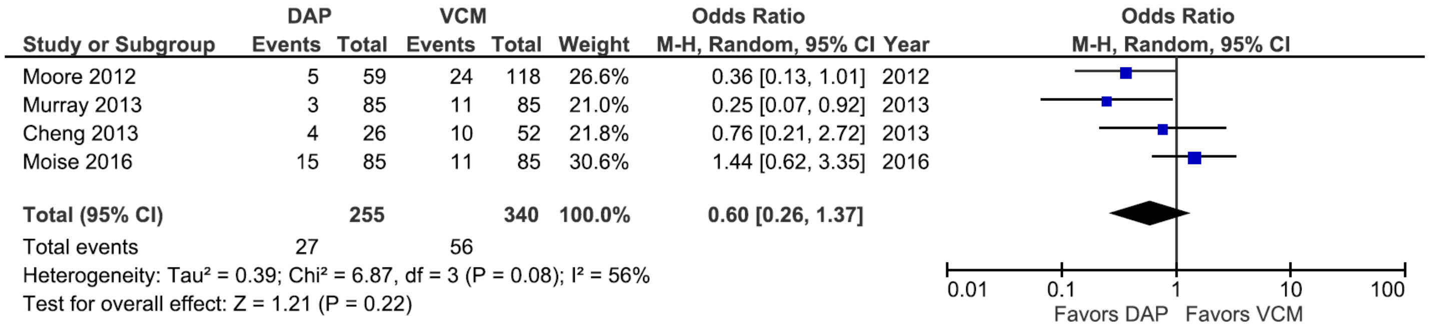

Figure S2. Forest plots of the mortality rates of patients treated with DAP *versus* VCM for bacteremia caused by MRSA with VCM MIC > 1 μg/mL determined by E-test or Microscan. Symbols and abbreviations are as described for Figure 2.

(A)

Study or Subgroup	DAP Events	DAP Total	VCM Events	VCM Total	Weight	Odds Ratio M-H, Random, 95% CI	Year
Moore 2012	2	59	6	118	54.4%	0.65 [0.13, 3.35]	2012
Murray 2013	0	85	3	85	16.3%	0.14 [0.01, 2.71]	2013
Cheng 2013	0	26	4	52	16.5%	0.20 [0.01, 3.92]	2013
Moise 2016	0	85	0	85		Not estimable	2016
Kalimuddin 2018	0	7	1	7	12.8%	0.29 [0.01, 8.39]	2018
**Total (95% CI)**		**262**		**347**	**100.0%**	**0.38 [0.11, 1.26]**	
Total events	2		14				

Heterogeneity: Tau² = 0.00; Chi² = 1.09, df = 3 (P = 0.78); I² = 0%
Test for overall effect: Z = 1.59 (P = 0.11)

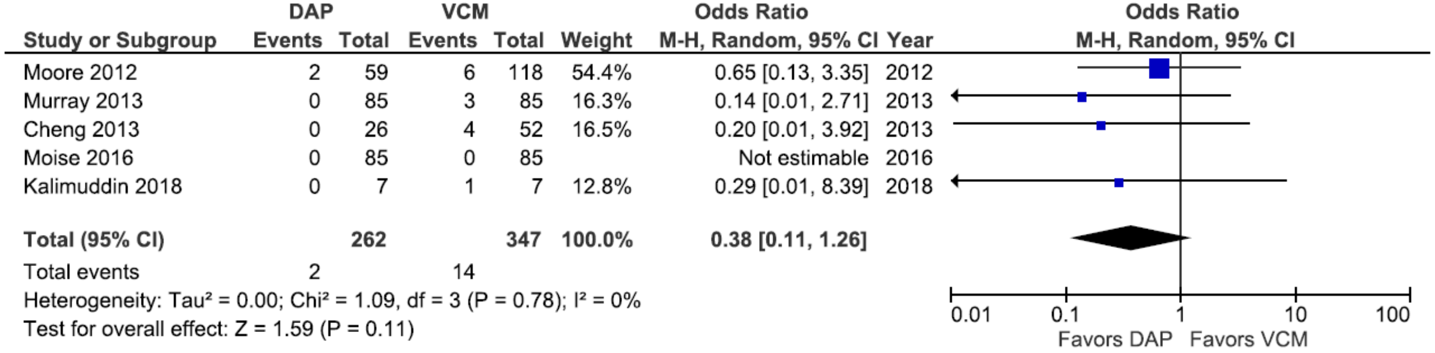

(B)

Study or Subgroup	DAP Events	DAP Total	VCM Events	VCM Total	Weight	Odds Ratio M-H, Random, 95% CI	Year
Moore 2012	22	78	21	82	53.2%	1.14 [0.57, 2.30]	2012
Moise 2016	16	85	19	85	46.8%	0.81 [0.38, 1.70]	2016
**Total (95% CI)**		**163**		**167**	**100.0%**	**0.97 [0.58, 1.61]**	
Total events	38		40				

Heterogeneity: Tau² = 0.00; Chi² = 0.45, df = 1 (P = 0.50); I² = 0%
Test for overall effect: Z = 0.12 (P = 0.91)

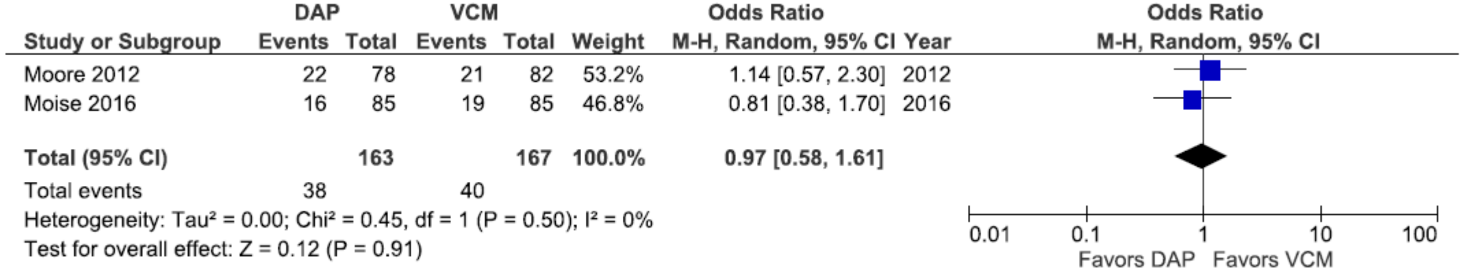

Figure S3. Forest plots of the recurrence and rehospitalization rates for patients treated with DAP *versus* VCM for bacteremia caused by MRSA with VCM MIC > 1 μg/mL.

(A and B) Forest plots of the recurrence rate (A) and rehospitalization rate (B) of patients treated with DAP *versus* VCM in the studies evaluated. Symbols and abbreviations are as described for Figure 2.

Table S1. Terms used and results of searches in PubMed.

No.	Search terms	Results
1	staphylococcus aureus	122068
2	methicillin-resistant staphylococcus aureus	32680
3	acute bacterial skin and skin structure infection	2044
4	skin and skin structure infection	138266
5	complicated skin and skin structure infection	38198
6	skin and soft tissue infection	7114
7	complicated skin and soft tissue infection	2467
8	uncomplicated skin and soft tissue infection	162
9	endocarditis	39924
10	infective endocarditis	39921
11	infectious endocarditis	8822
12	bacterial endocarditis	24600
13	bacteremia	46225
14	bacteraemia	46225
15	bacillaemia	25
16	bacillemia	42
17	bloodstream infection	29070
18	daptomycin	3235
19	lipopeptide	18679
20	cubicin	3236
21	vancomycin	30544
22	teicoplanin	4074
23	teichomycin	4075
24	teicomycn	1

25	targocid	4071
26	glycopeptide	64027
27	(#1) OR (#2) OR (#3) OR (#4) OR (#5) OR (#6) OR (#7) OR (#8) OR (#9) OR (#10) OR (#11) OR (#12) OR (#13) OR (#14) OR (#15) OR (#16) OR (#17)	343005
28	(#18) OR (#19) OR (#20)	19787
29	(#21) OR (#22) OR (#23) OR (#24) OR (#25) OR (#26)	79556
30	(#27) AND (#28) AND (#29)	1799

Table S2. Terms used and results of searches in Web of Science.

No.	Search terms	Results
1	staphylococcus aureus	137973
2	methicillin-resistant staphylococcus aureus	29579
3	acute bacterial skin and skin structure infection	610
4	skin and skin structure infection	3819
5	complicated skin and skin structure infection	940
6	skin and soft tissue infection	6830
7	complicated skin and soft tissue infection	1010
8	uncomplicated skin and soft tissue infection	213
9	endocarditis	34840
10	infective endocarditis	12868
11	infectious endocarditis	2774
12	bacterial endocarditis	7243
13	bacteremia	40130
14	bacteraemia	40130

15	bacillaemia	34
16	bacillemia	81
17	bloodstream infection	12381
18	daptomycin	4043
19	lipopeptide	4279
20	cubicin	65
21	vancomycin	33916
22	teicoplanin	4180
23	teichomycin	41
24	teicomycn	0
25	targocid	5
26	glycopeptide	8784
27	(#1) OR (#2) OR (#3) OR (#4) OR (#5) OR (#6) OR (#7) OR (#8) OR (#9) OR (#10) OR (#11) OR (#12) OR (#13) OR (#14) OR (#15) OR (#16) OR (#17)	210846
28	(#18) OR (#19) OR (#20)	7895
29	(#21) OR (#22) OR (#23) OR (#24) OR (#25) OR (#26)	40849
30	(#27) AND (#28) AND (#29)	2071

Table S3. Terms used and results in searches of the Cochrane Library.

No.	Search terms	Results
1	staphylococcus aureus	3343
2	methicillin-resistant staphylococcus aureus	1149
3	acute bacterial skin and skin structure infection	218
4	skin and skin structure infection	673

5	complicated skin and skin structure infection	234
6	skin and soft tissue infection	703
7	complicated skin and soft tissue infection	205
8	uncomplicated skin and soft tissue infection	35
9	endocarditis	602
10	infective endocarditis	372
11	infectious endocarditis	69
12	bacterial endocarditis	305
13	bacteremia	2460
14	bacteraemia	2459
15	bacillaemia	1
16	bacillemia	1
17	bloodstream infection	1055
18	daptomycin	187
19	lipopeptide	127
20	cubicin	19
21	vancomycin	1988
22	teicoplanin	302
23	teichomycin	0
24	teicomycn	0
25	targocid	7
26	glycopeptide	268
27	(#1) OR (#2) OR (#3) OR (#4) OR (#5) OR (#6) OR (#7) OR (#8) OR (#9) OR (#10) OR (#11) OR (#12) OR (#13) OR (#14) OR (#15) OR (#16) OR (#17)	7462
28	(#18) OR (#19) OR (#20)	303

29	(#21) OR (#22) OR (#23) OR (#24) OR (#25) OR (#26)	2307
30	(#27) AND (#28) AND (#29)	105

Table S4. Terms used and results in searches of ClinicalTrials.gov.

No.	Search terms	Results
1	staphylococcus aureus	389
2	methicillin-resistant staphylococcus aureus	183
3	skin and skin structure infection	179
4	skin and soft tissue infection	66
5	endocarditis	98
6	bacteraemia	346
7	bloodstream infection	188
8	daptomycin	81
9	lipopeptide	13
10	vancomycin	270
11	teicoplanin	19
12	glycopeptide	19
13	(#1) OR (#2) OR (#3) OR (#4) OR (#5) OR (#6) OR (#7)	1050
14	(#8) OR (#9)	94
15	(#10) OR (#11) OR (#12)	299
16	(#13) AND (#14) AND (#10)	15
17	(#13) AND (#14) AND (#11)	1
18	(#13) AND (#14) AND (#12)	0

Table S5. Adverse drug reactions with DAP and VCM in selected studies.

**Adverse Events**	Moore 2012		Murray 2013		Claeys 2016		Moise 2016		Cubist 2018		Kalimuddin 2018	
	DAP (n=59)	VCM (n=118)	DAP (n=85)	VCM (n=85)	DAP (n=131)	VCM (n=131)	DAP (n=85)	VCM (n=85)	DAP (n=19)	VCM (n=17)	DAP (n=7)	VCM (n=7)
Serious adverse events	—	—	—	—	—	—	—	—	3 (15.8%)	4 (23.5%)	3 (42.9%)	4 (57.1%)
CPK elevation	1 (1.7%)	—	1 (1.2%)	—	3*1 (2.3%)	0 (0.0%)	2 (2.4%)	0 (0.0%)	2 (10.5%)	0 (0.0%)	1 (14.3%)	0 (0.0%)
Nephrotoxicity	—	13/63*2 (20.6%)	—	22 (25.9%)	0 (0.0%)	12 (9.2%)	6/64*2 (9.4%)	14/62*2 (22.6%)	0 (0.0%)	4 (23.5%)	1 (14.3%)	0 (0.0%)
Dermatologic	—	—	—	—	—	—	—	—	0 (0.0%)	4 (23.5%)	0 (0.0%)	3 (42.9%)
Gastrointestinal	—	—	—	—	—	—	—	—	7 (36.8%)	3 (17.6%)	1 (14.3%)	0 (0.0%)

CPK, creatinine phosphokinase; DAP, daptomycin; VCM, vancomycin.

*1 Included two patients with myopathy.

*2 Patients on intermittent dialysis treatment and patients with acute kidney injury at baseline were excluded from the analysis.
